# Coupled online sequential extreme learning machine model with ant colony optimization algorithm for wheat yield prediction

**DOI:** 10.1038/s41598-022-09482-5

**Published:** 2022-03-31

**Authors:** Mumtaz Ali, Ravinesh C. Deo, Yong Xiang, Ramendra Prasad, Jianxin Li, Aitazaz Farooque, Zaher Mundher Yaseen

**Affiliations:** 1 Deakin-SWU Joint Research Centre on Big Data, School of Information Technology, Deakin University, Geelong, VIC 3125 Australia; 2 School of Agricultural, Computational and Environmental Sciences, International Centre for Applied Climate Sciences, Institute of Agriculture and Environment, University of Southern Queensland, Springfield, QLD 4300 Australia; 3 Department of Science, School of Science and Technology, The University of Fiji, Saweni, Lautoka Fiji; 4 Faculty of Sustainable Design Engineering, University of Prince Edward Island, Charlottetown, PE C1A4P3 Canada; 5 School of Climate Change and Adaptation, University of Prince Edward Island, Charlottetown, PE Canada; 6 Adjunct Research Fellow, USQ’s Advanced Data Analytics Research Group, School of Mathematics Physics and Computing, University of Southern Queensland, Toowoomba, QLD 4350 Australia; 7 New Era and Development in Civil Engineering Research Group, Scientific Research Center, Al-Ayen University, Thi-Qar, 64001 Iraq; 8 Institute for Big Data Analytics and Artificial Intelligence (IBDAAI), Universiti Teknologi MARA, Kompleks Al-Khawarizmi, 40450 Shah Alam, Selangor Malaysia

**Keywords:** Computer science, Ecological modelling, Ecosystem ecology

## Abstract

Inadequate agricultural planning compounded by inaccurate predictions results in an inflated local market rate and prompts higher importation of wheat. To tackle this problem, this research has designed two-phase universal machine learning (ML) model to predict wheat yield (W_pred_), utilizing 27 agricultural counties’ data within the Agro-ecological zone. The universal model, online sequential extreme learning machines coupled with ant colony optimization (ACO-OSELM) is developed, by incorporating the significant annual yield data lagged at (*t* − 1) as the model’s predictor to generate future yield at 6 test stations. In the first phase, ACO is adopted to search for suitable, statistically relevant data stations for model training, and the corresponding test station by virtue of a feature selection strategy. An annual wheat yield time-series input dataset is constructed utilizing data from each selected training station (1981–2013) and applied against 6 test stations (with each case modelled with 26 station data as the input) to evaluate the hybrid ACO-OSELM model. The partial autocorrelation function is implemented to deduce statistically significant lagged data, and OSELM is applied to generate W_pred_. The two-phase hybrid ACO-OSELM model is tested within the 6 agricultural districts (represented as stations) of Punjab province, Pakistan and the results are benchmarked with extreme learning machine (ELM) and random forest (RF) integrated with ACO (i.e., hybrid ACO-ELM and hybrid ACO-RF models, respectively). The performance of the ACO-OSELM model was proven to be good in comparison to ACO-ELM and ACO-RF models. The hybrid ACO-OSELM model revealed its potential to be implemented as a decision-making system for crop yield prediction in areas where a significant association with the historical agricultural crop is well-established.

## Introduction

Adoption of new knowledge about the best approaches to farming and strategic crop management systems, whilst learning the best practices from neighbourhood cropping zones, are considered as useful tools^1–3^. Agronomists use this technique to formulate precise and suitable evidence on future crop yield and bring benefits to the farmers^4–6^. In Pakistan, wheat is one of the most commonly grown crops^7^. Wheat contributes to up to 2.6% of Pakistan’s gross domestic product (GDP) while considering agronomy division its contribution is 12.5% of the GDP^8^. According to the United Nations, Pakistan was ranked in the top eight global wheat producers between 2016 and 2019^9,10^. Wheat is produced during the winter period, largely in the province of Punjab^11^.

The current wheat yield prediction and forecasting methods adopted by the Pakistan Government have been reported to be highly inaccurate^12^. In 2005, poor wheat yield predictions of Pakistan where the actual production was relatively small equated to the estimated yield, resulted in an inflated local market rate and prompted higher importation of wheat^13,14^. Similarly, in 2012–2013, Pakistan experienced severe challenges in wheat supply, which happened due to the lower production of wheat yield in Punjab^15^. A plausible reason for this deficit was attributed to poor agricultural planning and inaccurate predictions to satisfy the national grain needs. Sajjad reported about the looming wheat scarcities for an agriculturally rich country, Pakistan^16^. Due to these concerns that have a direct detrimental impact on income and food security for the already staggering economy of developing Pakistan, the government representatives work towards enhancing the forecast to account for the surplus and shortfalls in advance.

The modelling of wheat yield utilizing ancient procedures and irrelevant information from past yields is bound to deter the outcomes drastically^17,18^. To calculate the future productions, establishing novel systems for improving current and future agricultural productions, and supporting future food security issues in both developing and first-world nations are necessary. This validates the essential role of wheat yield modelling via novel artificial intelligence (AI) modelling or data-intelligent approaches that encapsulate relevant historical patterns. Data-intelligent techniques have enormous flexibility in crop management due to their ease of employment, viable accuracy, and feature detection capabilities^19–22^. These models are also bound to empower officials in attaining efficient ways to predict future crop productions^23^. There are several examples of data intelligent algorithms in agronomy. Dempewolf et al. implemented vegetation index to predict wheat yield in Punjab^24^, whereas Hamid examined the wheat frugality and future forecasts^25^. On the other hand, Muhammad investigated the historic context of the wheat improvements for Balochistan^26^. Furthermore, Iqbal et al. designed an auto-regressive integrated moving average model (ARIMA) to predict future wheat and yields up to the year 2022 in Pakistan^27^. Sher and Ahmad integrated the Cobb–Douglas function with ARIMA to predict wheat yield^28^. However, the works have constructed simplistic regression models (e.g., ARIMA) that are often discredited due to their assumptions of linearity in the data^29^.

Based on previous approaches for wheat yield prediction, Rahman et al. developed a data-driven approach to predict rice yield for Bangladesh^30^, whereas monitoring of rice crop was implemented via a neural network model^31^. Similarly, an artificial neural network (ANN) was developed for soybean and corn predictions in Malaysia^32^. In addition, all the forgoing works were on a provincial level, or a country-wide, which lacks the significance to a small locality such as the district level forecasting used in this study for better accuracy and applicability. Yield prediction is a challenging job as various interconnected climatic drivers affect the yield^5^. Thus, agronomic experts can possibly use the preceding yields to predict future production. Despite this, none of the previous work has utilized wheat yield of several locations for training purposes to predict the yield of other stations.

Information from several other locations for training purposes to predict the yield at the main region is essentially beneficial in decision support systems since it can allow the modellers to accept analogous features prevailing to be analysed to evaluate the main region data^33^. This framework can be adopted in agricultural practices by associating station-specific crop production and creating suitable deductions relevant to the existence of favourable (or unfavourable) eco-friendly or soil fertility circumstances to produce maximum yield^34^. Considering the need for accurate future wheat yield prediction, the modelling of crop yield using several stations yield data for model development can offer a reasonable system to determine the most cost-effective and useful agricultural monitoring practices.

The proposed two-phase AI system called (i.e., ACO-OSELM) model was adopted in the current research to predict wheat yield. Two benchmark models including random forest (RF) and extreme learning machine (ELM) and their hybrid versions were designed for verification of the ACO-OSELM model. ELM and RF models were selected as a benchmark due to their remarkable predictive potentials as appear in the literature^35–39^. The selection of the OSELM was owing to the main merit of the ELM model^40^. ELM model is a single layer feed-forward neural network (SLFN) where the input weights are randomly assigned while the output weights are analytically determined^41^. Unlike conventional neural network models, the ELM is able to avoid issues such as tuning of learning rates, learning epochs, stopping criteria and local optima making it computationally efficient^42^. In addition, ELM efficiently handles large-scale data with a better generalization capability and is more suited to large-scale wheat yield predictions^43,44^. On the other hand, the RF models are ensemble regression tree models that use the bootstrap aggregation (i.e., bagging) approach to generate forecasts^45–47^. The RF model ameliorates the overfitting issue, which is a key drawback of conventional solitary regression tree-based models^48^. Consequently, these models have been applied in this study. The two-phase hybrid ACO-OSELM is validated for wheat yield prediction in agronomic regions: Rahimyar Khan, Dera Ghazi Khan (denoted as D. G. Khan), Kasur, Sialkot, Rawalpindi, and Jhang located in Punjab province, Pakistan where 26 stations from 26 districts were used to develop the model. The selected study stations are spread throughout the Punjab province and are the major wheat producers. These stations are chosen randomly from the agriculturally rich Punjab province. To verify the applicability of the proposed ACO-OSELM model, this study aims to fulfill three objectives: (i) To develop a bio-inspired ACO algorithm to select the best possible neighbouring stations located in Punjab province, Pakistan for training purposes using feature selection strategy; (ii) To incorporate the statistically important one step antecedent data (i.e., *t* − 1 where *t* represents the current data) of the selected training stations in the OSELM model to develop a two-phase hybrid ACO-OSELM model to predict the current and future wheat yield; and (iii) To assess the predictive accuracy of the two-phase ACO-OSELM model for wheat yield prediction universally in the whole province of Punjab in Pakistan.

## Theoretical overview

The architecture involved in the establishing of a two-phase hybrid ACO-OSELM model for wheat yield prediction is discussed here.

### Ant colony optimization (ACO) algorithm

Dorigo and Di Caro^49^ presented the ACO feature selection procedure, which has been widely used in different applications^50–53^. This study utilized the ACO technique to determine the least possible distance between wheat yield (W) of the training stations, and the testing stations, a strategy that can be adopted to choose the respective training stations for yield prediction at the testing station. A parameter named pheromone in the ACO process is allotted to predictor stations, which categorizes these predictors alongside the target/test stations at the start. The trial pheromone value is used to compute the probability of the chosen station to train against the test station while the magnitude pheromone alters by navigating the training stations, and subsequently, the probability is improved for the next coming ants to pick the optimum station. Readers can survey the following literature for more details on the ACO procedure experiment^54,55^.

### Extreme learning machine (ELM)

Huang et al.^42^ designed a fast machine learning model consisting of Single Layer Feedforward Neural Network (SLFN) called the ELM that is computationally far more efficient^56^. In mathematical terms, the ELM can be expressed as:

1 $$\sum _{i=1}^{N}{\rho }_{i}{f}_{i}\left(x\right)={W}_{for}\left(x\right)$$

where $\rho = {\left[{\rho }_{1}, {\rho }_{2},\dots ,{\rho }_{M}\right] }^{T}$ is the output weight vector between the hidden layer of M nodes to the m ≥ 1 output nodes, and $f\left(x\right)= {\left[{f}_{1}\left(x\right), {f}_{2}\left(x\right),\dots ,{f}_{N}\left(x\right)\right] }^{T}$ is ELM nonlinear feature mapping and ${W}_{for}\left(x\right)$ is the final output/prediction. The function ${W}_{for}\left(x\right)$ denotes the predicted wheat yield (*W*) at the $i{\text{th}}$ hidden node. Various output functions may be applied in different hidden neurons. For instance:

2 $${f}_{i}\left(x\right)=G\left({a}_{i},{b}_{i},x\right),\,\, {a}_{i}\in {R}^{d},\,\, {b}_{i}\in R$$

The term $G\left(a,b,x\right)$ is representing a nonlinear piecewise continuous function satisfying ELM universal approximation capability theorems^57^, (a, b) are the hidden node parameters and $R$ is the set of real numbers whereas ${R}^{d}$ is the *d*-dimensional set of real numbers and $x$ is the input data. The activation functions are Sigmoid, Hyperbolic tangent, Gaussian, Hard limit, Cosine and Fourier basis functions.

Initially, ELM randomly modifies the hidden layer to project the inputs into a feature space using some piecewise continuous nonlinear functions^58^. The parameters (a, b) are generated randomly that are not dependent on the training set. In the second phase of ELM learning, then, the weights $(\rho )$ linking the hidden and the output layer are solved by minimizing the prediction error in the squared error sense: i.e.

3 $$\begin{array}{c}min\\ {\rho \in R}^{N\times n}\end{array}{\Vert M\rho -T\Vert }^{2}$$

where $M$ is denoting the hidden layer output matrix and $T$ is the training data matrix which can be simplified as follows^57^. The ∥ · ∥ indicates the Frobenius norm.

4 $$M=\left[\begin{array}{c}m\left({x}_{1}\right)\\ \vdots \\ m\left({x}_{N}\right)\end{array}\right]={\left[\begin{array}{ccc}{m}_{1}\left({x}_{1}\right)& \cdots & {m}_{N}\left({x}_{1}\right)\\ \vdots & \cdots & \vdots \\ {m}_{1}\left({x}_{N}\right)& \cdots & {m}_{N}\left({x}_{N}\right)\end{array}\right]}$$

5 $$T=\left[\begin{array}{c}{{t}_{1}}^{T}\\ \vdots \\ {{t}_{N}}^{T}\end{array}\right]={\left[\begin{array}{ccc}{t}_{11}& \cdots & {t}_{1n}\\ \vdots & \cdots & \vdots \\ {t}_{N1}& \cdots & {t}_{Nn}\end{array}\right]}$$

The ideal solution to (3) is provided by:

6 $${\rho }^{*}={M}^{+}T$$

In Eq. (6) ${M}^{+}$ is indicates the Moore–Penrose generalized inverse of *M*. The SLFNs with randomly chosen input weights successfully learn various training patterns with the least error. In this way, SLFNs can be considered as a linear system. The output weights which attach the hidden layer to the output layer in the linear system can now be analytically solved by generalized inverse operation of the hidden layer output matrices. Thus, the ELM model is faster than the conventional feedforward learning algorithms^59,60^.

### Online-sequential extreme learning machine (OSELM)

In OSELM the data is channelled in a chunk-by-chunk manner for better understanding and accuracy, whereas in ELM a total of *N* training data points are used for training purposes, which becomes computationally time exhaustive further affecting the learning procedure^61^. Therefore, the OSELM, which is an advanced form of ELM, operates in two learning stages utilizing the chunk-by-chunk approach i.e., initialization followed by the sequential learning stage. In the initialization phase, the *H* matrix is packed like ELM, for later usage. The randomized weights together with the biases are allocated to respective chunks of primary wheat yield (W) data to determine the output matrix of the OSELM hidden layers. Then the sequential learning stage is launched either in a one-by-one manner or a lump-by-lump fashion where the one-time data utilization is not permissible. More specific information on OSELM can be found in the following (e.g.,^62–64^). For a given training set ${\beth }_{k-1}$ in the initialization phase:

7 $${\beth }_{k-1}=\left\{\left({x}_{j},{t}_{j}\right):j=1,2,\dots k-1\right\}$$

The term ${\beth }_{k-1}$ indicates the training dataset whereas ${x}_{j}$ is the input data and ${t}_{j}$ is the *j*th parameter. The first output weight is provided by the following equation:

8 $${\rho }_{k-1}={\varnothing }_{k-1}{M}_{k-1}^{t}{T}_{k-1}$$

The term ${\rho }_{k-1}$ is showing the initial output weight, ${\varnothing }_{k-1}={\left({M}_{k-1}^{t}{M}_{k-1}\right)}^{-1}$ is indicating the Moore–Penrose generalized inverse of the matrix, ${{M}_{k-1}=\left[{m}_{1}^{t}, \dots ,{m}_{k-1}^{t}\right]}^{t}$ is denoting the hidden layer output matrix, and ${{t}_{k-1}=\left[{t}_{1}, \dots ,{t}_{k-1}\right]}^{t}$ is the training data matrix. The biases and random weights are allocated in a small chunk in the initialization stage to calculate the hidden layer output matrix in the initial wheat yield (W) training data.

The sequential learning phase is then commenced where RLS algorithm is utilized to modify the output weights in a recursive way^61^. The output weights in OSELM are recursively updated based on the intermediate results in the last iteration and the newly arrived data, which is deleted immediately once the features have been learnt, and therefore, the calculation overhead and the memory requirement of the algorithm are significantly decreased.

### Random forest (RF)

The RF model is a regression tree-based learning approach whereby the bootstrapping and bagging are the underpinning modeling techniques on which the RF ensemble modeling approach is constructed upon^65,66^. Using a random bagging technique, the RF model develops ensembles in which each node is linked randomly by choosing the relevant inputs for better efficiency while avoiding overfitting^67^. The subsequent stages provide a brief account of the RF model designing:

i. Perform bootstrapping on the input predictors to create *n*-bootstrapped based trees (i.e., *n*_trees_).
ii. Determine the largest no. of split variables (*m*_*try*_) by means of random sampling with a non-prune regression tree.
iii. Aggregate the simulated *n*_trees_ to predict wheat yield (W).

The RF algorithm has been used in many applications including water quality^46^, soil moisture^68^, ecological^69^, hydrological^70^ and solar radiation^71,72^ forecasting applications.

## Case study description and data

### Study region and wheat yield data

Pakistan’s Federal Bureau of Statistics and the Agriculture Marketing Information Services, Directorate of Agriculture provided the wheat yield data (Economics & Marketing)^73,74^. The study stations included are of high agricultural significance for the Punjab province, Pakistan. In this paper, each district is represented as a station. Agricultural sectors in Punjab province play an important part with economic contributions of 56.1–61.5%^75^. Further, a widespread irrigation network enables this province's rich agricultural zone. Considering Punjab as a key agronomic belt, the adoption of AI techniques to predict wheat yield is an interesting research endeavour. To establish the time series wheat yield dataset, the district-level wheat production was assimilated. To construct this dataset, the areal (district level) productions of wheat were acquired through the provincial Crop Reporting Services which had been compiled by the Economic Wing of the devolved Ministry of Food and Agriculture, and later by the Pakistan Federal Bureau of Statistics. The acquired data had some missing wheat yield values for 2009. To overcome this issue, the average of all other data for the period 1981–2013 is used to recover the missing data of the predictor and the corresponding target stations.

**Figure 1:**
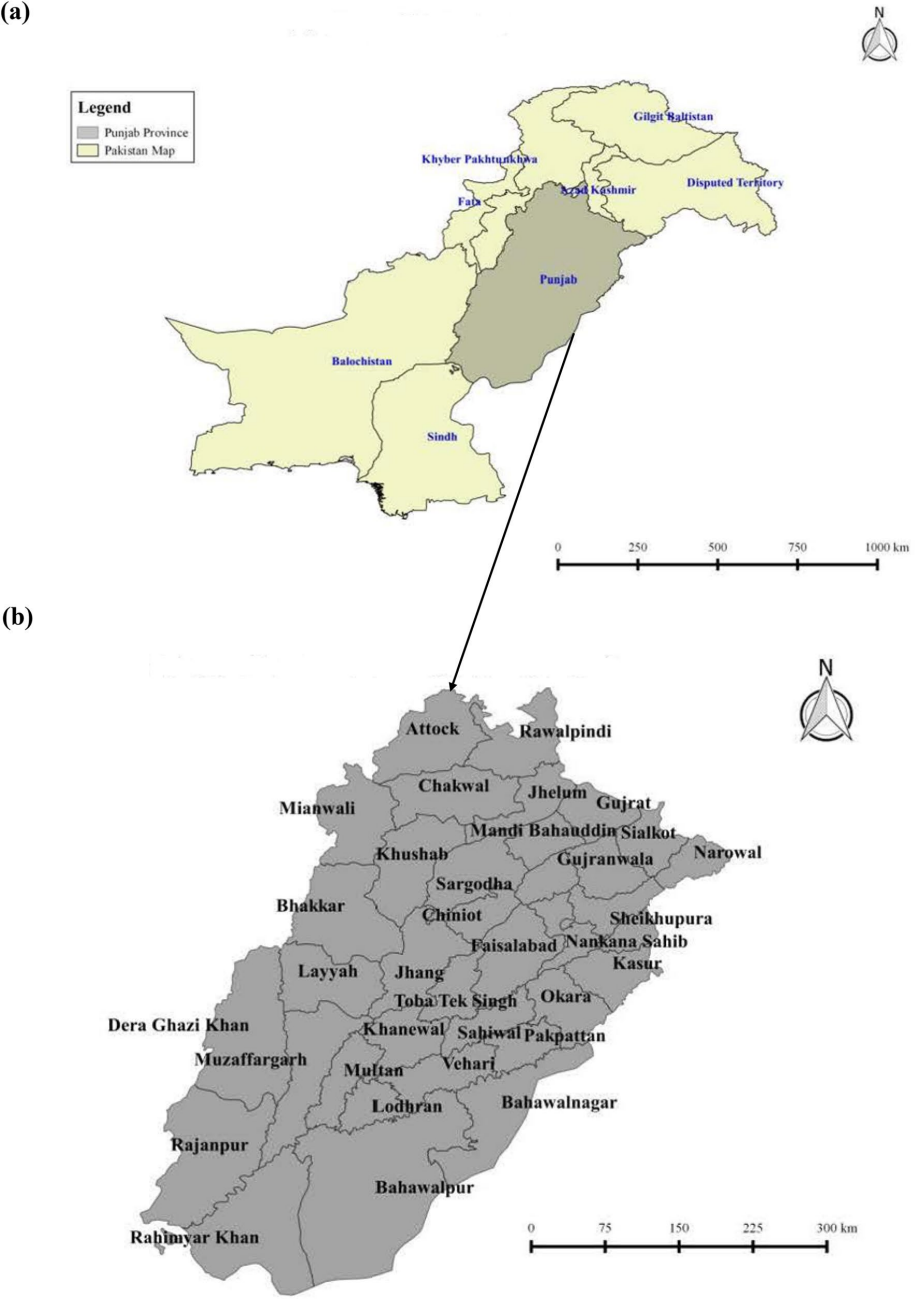
Map of the study region. (**a**) Provinces of Pakistan. (**b**) Districts of Punjab where the present study was undertaken.

**Figure 2:**
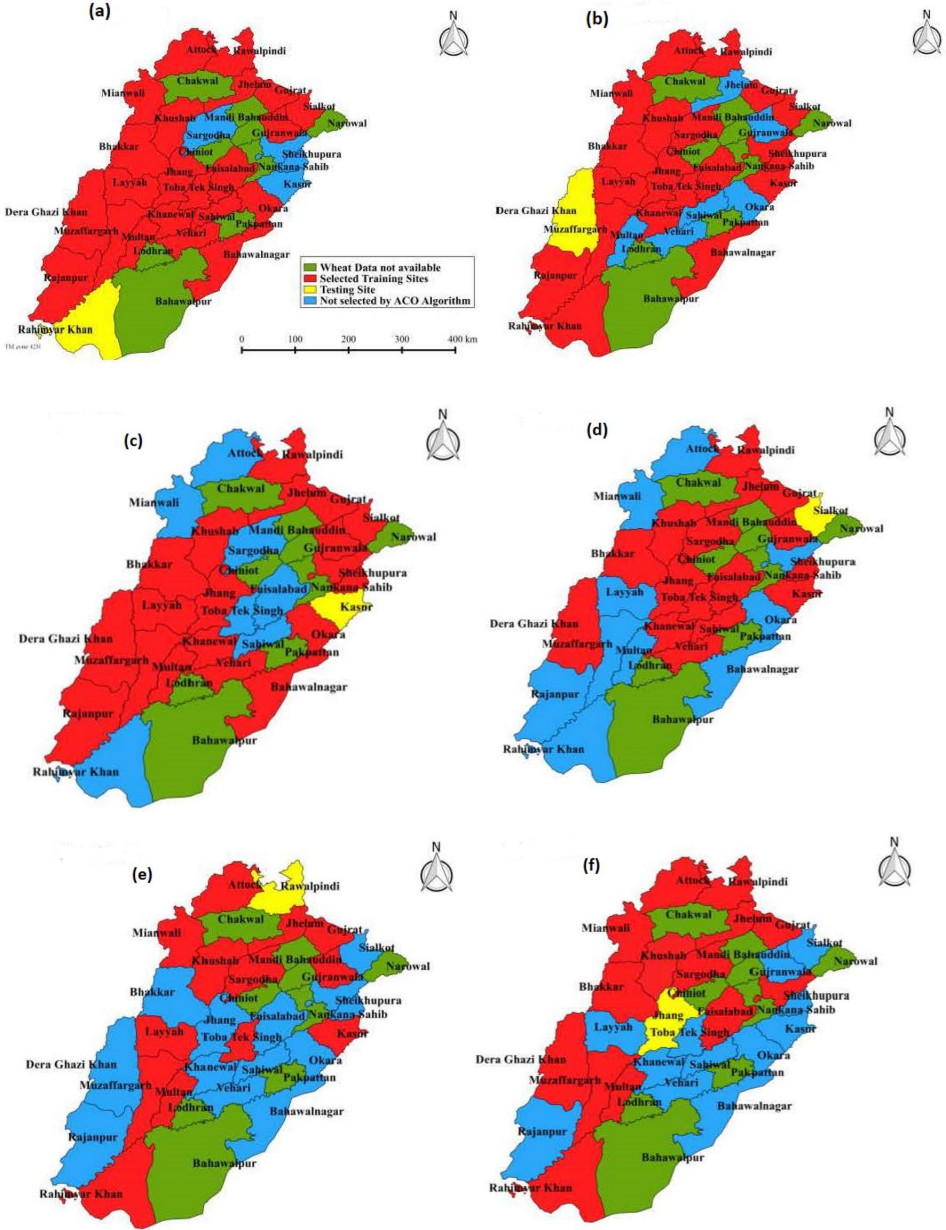
The selected training stations in red and the corresponding test stations in yellow are (**a**), (**b**), (**c**), (**d**), (**e**), and (**f**) respectively. Note that the stations shown in green have ‘no available wheat yield data’ and those in blue were not selected by the ant colony optimisation algorithm.

Figure 1a,b show the provinces in Pakistan and the map of all districts in Punjab province (current study region). The figure illustrates the study stations (i.e., the major districts) of wheat farming. Figure 2a–f present the total of 6 maps which represents the testing stations (yellow colour), training stations (red colour), and the stations where wheat yield data is not available (green colour) and the stations that are not selected by ACO algorithm (blue colour). Figures 1 and 2 were generated using the GIS software^76^. A total of 27 stations were considered with data from 1981 to 2013. To obtain the wheat yield time series, out of 27 stations, 26 stations were used for the selection of the best stations for training to develop the model in relation to the remaining (1) testing station. Each time, 26 stations were used to select the best stations for training subsets against the 6 test stations. Table 1 presents basic statistics (i.e., latitude, longitude, and elevations), maximum, minimum, standard deviation, skewness, and kurtosis) of the present study stations.

**Table 1:** Geographic properties and wheat yield statistics of the study stations for Punjab, Pakistan.

Stations	Geographic characteristics			Wheat yield statistics (kg ha^−1^)					
	Latitude (N)	Longitude (E)	Elevation (m)	Mean	Std.	Min.	Max.	Skewness	Kurtosis
Shekhupura	31.71°	73.98°	207	2297.46	519.54	1087.89	3062.99	− 0.29	− 0.65
Okara	30.81°	73.45°	105	2901.89	528.93	1888.08	3588.10	− 0.26	− 1.16
Sahiwal	30.66°	71.11°	152	2751.87	411.29	1955.17	3792.40	0.12	− 0.10
Vehari	30.04°	72.34°	140	2568.94	456.80	1846.00	3462.03	0.09	− 0.87
Multan	30.15°	71.52°	122	2307.97	377.77	1713.03	2952.97	0.16	− 1.17
Muzaffar Garh	30.07°	71.18°	122	2151.16	470.69	1157.47	2890.72	− 0.21	− 0.99
Dera Ghazi Khan	30.03°	70.38°	129	2234.18	457.67	1050.86	2914.07	− 0.66	0.32
Bakar	45.30°	14.53°	82	2049.52	601.05	1222.01	3440.61	0.79	0.18
Layyah	30.96°	70.94°	143	2025.44	485.42	1160.59	2900.23	0.21	− 0.88
Khushab	32.32°	71.90°	195	1455.08	345.98	822.00	2103.04	0.09	− 0.88
Sargodha	32.08°	72.66°	189	2253.13	308.08	1637.40	2742.39	0.02	− 1.06
Faisalabad	31.45°	73.13°	184	2487.44	511.38	1565.83	3257.58	− 0.07	− 1.35
Toba Tek Singh	30.97°	72.48°	149	2757.25	715.26	1814.86	3310.60	2.20	5.95
Gujrat	32.57°	74.07°	233	1632.04	267.08	996.51	1985.37	− 0.77	− 0.03
Rawalpindi	33.56°	73.01°	508	1374.83	372.15	624.01	1993.06	− 0.26	− 0.68
Jhelum	32.74°	73.72°	234	1418.49	365.23	752.09	2110.03	− 0.01	− 0.64
Mianwali	32.58°	71.53°	210	1618.80	332.99	1037.09	2510.39	0.63	0.28
Lahore	31.52°	74.35°	217	2485.52	431.22	1385.93	3209.78	− 0.97	1.08
Khanewal	30.28°	71.93°	128	2613.95	436.54	1850.94	3600.46	0.24	− 0.67
Rajanpur	29.10°	70.32°	97	2138.37	512.46	1009.88	3012.48	− 0.70	− 0.10
Bahawal Nagar	30.00°	73.24°	163	2298.80	544.52	1401.50	3773.33	0.57	0.08
Attock	33.76°	72.36°	358	1271.96	326.45	685.73	2029.07	0.28	− 0.36
Gujranwala	32.15°	74.18°	229	2437.28	604.31	1055.69	3484.13	− 0.29	− 0.56
Jhang	31.30°	72.32°	158	2353.19	431.16	1637.83	3089.02	0.03	− 1.06
Kasur	31.11°	74.44°	218	2495.00	407.78	1688.95	3099.40	− 0.07	− 0.93
Rahimyar Khan	28.42°	70.29°	80	2308.74	522.80	1312.27	3369.46	0.29	− 0.49
Sialkot	32.49°	74.52°	256	2051.86	612.39	598.89	3018.60	− 0.44	− 0.47

### Design of two-phase hybrid ACO-OSELM model

The two-phase hybrid ACO-OSELM model was developed on MATLAB R2016b platform, (The Math Works Inc. USA) with Pentium 4 dual-core Central Processing Unit (CPU). To develop the proposed two-phase universal ACO-OSELM model, historical wheat yield data series were used. In this paper, *W* represents the wheat yield, *W*_*obs*_ denotes the rvobserved wheat yield while *W*_*pred*_ represents the predicted wheat yield. The original wheat yield data that had statistically significant lagged values at (*t* *−* 1) were employed as the input predictors in the first phase of the developmental stages. The development of the two-phase hybrid ACO-OSELM model involved the following phases:

#### Phase 1

In the foremost phase, the selected stations for model training were determined using the robust ACO feature selection strategy. Then, the user-defined parameters were defined with the ant numbers as 10 having 20 iterations and the initial pheromone factor was 1. For every station, the number of predictor stations (features) was defined prior to running the model. For Rahimyar Khan, the number of selected feature stations was 22, D. G. Khan (20), Kasur (19), Sialkot (17), Rawalpindi (12) and Jhang (14). The selected training stations with their correlation *r* against testing stations are described in Table 2.

**Table 2:** Selected training stations using ant colony optimization (ACO) algorithm with the correlation coefficient (*r*) for each training station against the testing station.

Test stations	ACO selected training stations	Correlation (*r*)	Test stations	ACO selected training stations	Correlation (*r*)	Test stations	ACO selected training stations	Correlation (*r*)
Station 1—Rahimyar Khan	Khanewal	0.855	Station 2—D. G. Khan	Rahimyar khan	0.781	Station 3—Kasur	Sialkot	0.926
	Faisalabad	0.723		Attock	0.310		Gujranwala	0.950
	Bahawal Nagar	0.854		Sialkot	0.779		Jhelum	0.592
	Multan	0.432		Sargodha	0.810		Layyah	0.817
	Gujranwala	0.817		Rajanpur	0.861		Rajanpur	0.664
	D. G. Khan	0.708		Jhang	0.764		Bakkar	0.837
	Khushab	0.638		Layyah	0.816		Bahawal Nagar	0.911
	Okara	0.644		Khushab	0.720		Vehari	0.942
	Vehari	0.659		Mianwali	0.643		Jhang	0.930
	Toba Tek Singh	0.556		Lahore	0.644		Lahore	0.406
	Rawalpindi	0.595		Toba Tek Singh	0.685		khanewal	0.805
	Sialkot	0.782		Shekhupura	0.791		Muzaffar Garh	0.858
	Sahiwal	0.602		Kasur	0.780		Okara	0.890
	Layyah	0.568		Bahawal Nagar	0.727		D. G. Khan	0.780
	Muzaffar Garh	0.778		Faisalabad	0.812		Multan	0.905
	Attock	0.628		Khanewal	0.704		Shekhupura	0.947
	Jhang	0.848		Bakkar	0.828		Gujrat	0.649
	Bakkar	0.841		Rawalpindi	0.333		Rawalpindi	0.443
	Rajanpur	0.360		Gujrat	0.509		Khushab	0.819
	Mianwali	0.341		Muzaffar Garh	0.881			
	Gujrat	0.480						
	Jhelum	0.185						
Stations 4—Sialkot	Faisalabad	0.420	Station 5—Rawalpindi	Muzaffar Garh	0.560	Station 6—Jhang	Rawalpindi	0.537
	D. G. Khan	0.425		Toba Tek Singh	0.801		D. G. Khan	0.764
	Sargodha	0.468		Mianwali	0.758		Multan	0.901
	Gujrat	0.183		Attock	0.876		Gujrat	0.722
	Khushab	0.382		Gujranwala	0.873		Jhelum	0.657
	Jhang	0.522		Multan	0.589		Khushab	0.873
	Vehari	0.479		Jhelum	0.944		Rahimyar khan	0.919
	Lahore	0.310		Sargodha	0.945		Attock	0.571
	Sahiwal	0.346		Khushab	0.907		Sargodha	0.906
	Gujranwala	0.530		Gujrat	0.781		Bakkar	0.842
	Jhelum	0.649		Kasur	0.898		Muzaffar Garh	0.855
	khanewal	0.489		Rahimyar khan	0.899		Mianwali	0.485
	Bakkar	0.370					Faisalabad	0.818
	Rawalpindi	0.181					Shekhupura	0.939
	Toba Tek Singh	0.571						
	Khanewal	0.782						
	Kasur	0.463						

**Figure 3:**
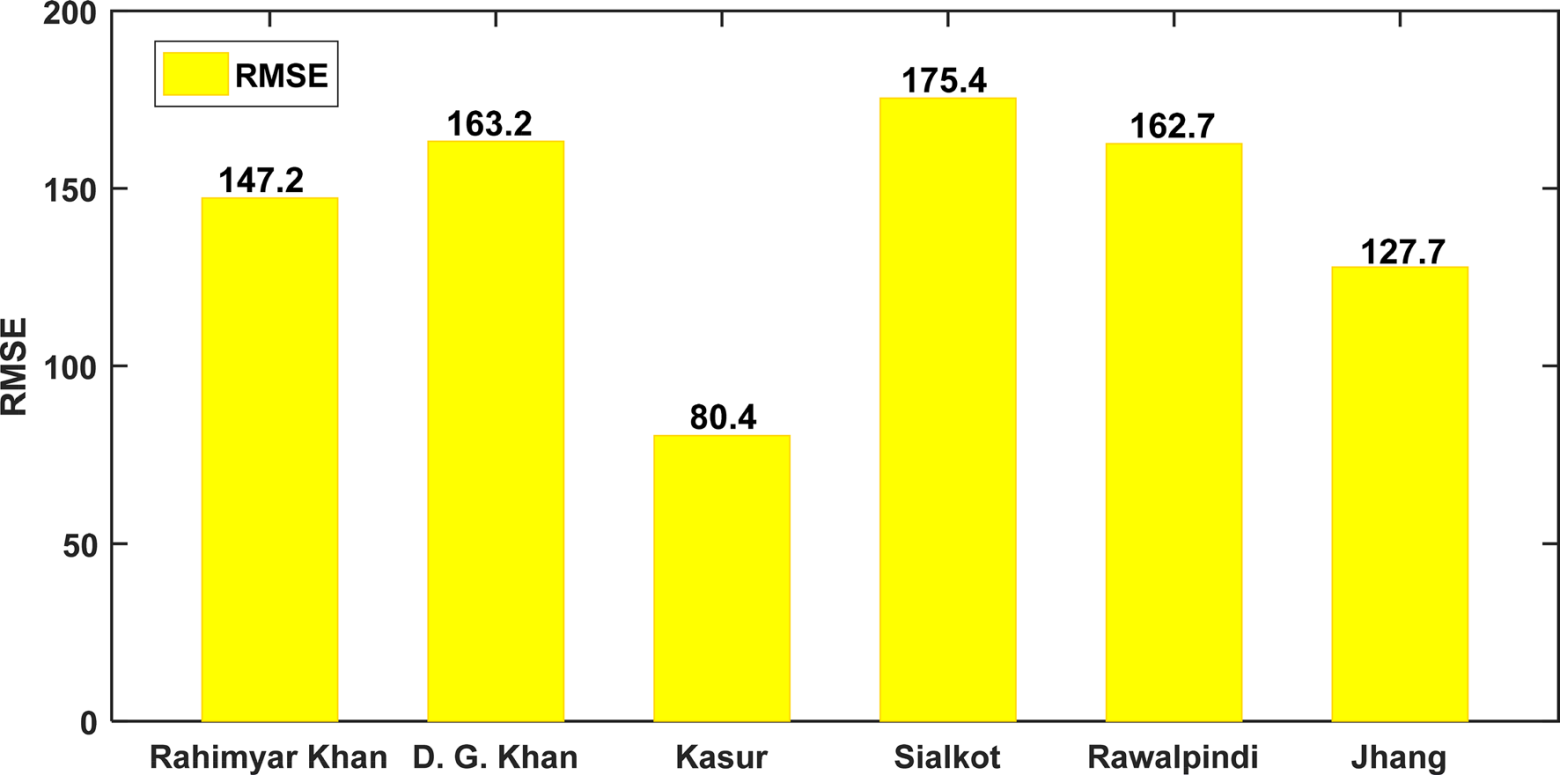
Bar graphs of the root mean squared error (RMSE) encountered by the ant colony optimisation algorithm in the selection of training study stations for each testing study station: Station 1: Rahimyar Khan, Station 2: D. G. Khan, Station 3: Kasur, Station 4: Sialkot, Station 5: Rawalpindi, and Station 6: Jhang.

The proposed two-phase hybrid ACO-OSELM model was trained and tested on a longer time series as well as at a shorter time series to assess the accuracy and universal performance of the model. This is to ensure that the model could be applied to other locations in Pakistan. In addition, a different number of surrounding predictor stations (features) were defined for every other testing station. Essentially, the Rahimyar Khan testing station had the largest number of surrounding training stations (i.e., 22) having the longest time series with 726 data points in the time series. On the other hand, Rawalpindi testing station has 12 training stations being selected with 396 data points being the shortest time-series used in the study. Table 3 shows the training data lengths for respective stations. In addition, the pheromone exponential weights and heuristic exponential weights were kept as 1. Figure 3 plots the *RMSE* errors that occurred in optimizing the cost and objective function of the ACO algorithm for identifying the best feature stations.

**Table 3:** Training data points (in terms of selected training stations) and testing data points for each testing station using the ACO algorithm.

Testing stations	No. of selected stations	The ratio of selected station	No. of data points in each station	No. of training data	No. of testing data	Skewness		Kurtosis		Standard deviation		Mean	
						Training	Testing	Training	Testing	Training	Testing	Training	Testing
Rahimyar Khan	22	0.846	33	22 × 33 = 726	33	0.026	0.290	− 0.660	− 0.491	640.03	522.80	2126.59	2308.74
D. G. Khan	20	0.769	33	20 × 33 = 660	33	− 0.023	− 0.661	− 0.639	0.322	604.39	457.67	2100.48	2234.18
Kasur	19	0.731	33	19 × 33 = 627	33	0.134	− 0.072	− 0.532	− 0.934	623.22	407.78	2101.54	2495.00
Sialkot	17	0.654	33	17 × 33 = 561	33	− 0.124	− 0.440	− 0.647	− 0.472	620.67	612.39	2193.14	2051.86
Rawalpindi	12	0.461	33	12 × 33 = 396	33	0.638	− 0.264	1.593	− 0.680	646.02	372.15	2008.08	1374.83
Jhang	14	0.538	33	14 × 33 = 462	33	0.233	0.033	− 0.537	− 1.061	588.81	431.16	1918.63	2353.19

The ratio of selected stations against testing stations, skewness, and kurtosis of training and testing data.

Since the data consisted of annual values from 1981 to 2013, which resulted in a total of 33 data records. ML models sometimes perform poorly on shorter time series. To handle this issue, we adopted the approach of selection of stations by ACO algorithm for training purposes and test the proposed model on the whole time-series data for respective testing stations. This technique of utilizing yield data from surrounding study stations to predict the yield of test stations are practically useful since it can enable the modellers to extract similar features and patterns prevalent at a predictor station to be analysed to estimate the yield at a testing station. This modelling approach does not required the splitting of the data in the traditional manner.

After carefully selecting the training stations for respective testing stations using the ACO algorithm, their correlation *r* (of selected training stations) against testing stations were calculated to confirm the linear relationship among them. For the study station Rahimyar Khan, the training station Khanewal registered the highest value of *r* ≈ 0.855, followed by Bahawal Nagar (*r* ≈ 0.854). Similarly, Muzaffar Garh (*r* ≈ 0.881) and Rajanpur (*r* ≈ 0.861) have the largest values of correlation with station D. G. Khan. For the study station, Kasur, Gujranwala, and Shekhupura attained the highest values of (*r* ≈ 0.950, 0.947). Table 2 summarizes all the correlation coefficients for respective stations. On the other hand, Station Kasur has the smallest cost to objective function *RMSE* followed by Jhang station (Fig. 3). Table 3 presents the number of datum points for training and testing purposes in each station with a ratio of selected stations against testing stations, with training and testing data distribution parameters. To prevent the differences in skewness in training and testing affecting the outcomes, data normalization was carried out using the following equation:

9 $${W}_{norm}=\frac{\left(W-{W}_{min}\right)}{\left({Wmin}_{max}\right)}$$

**Figure 4:**
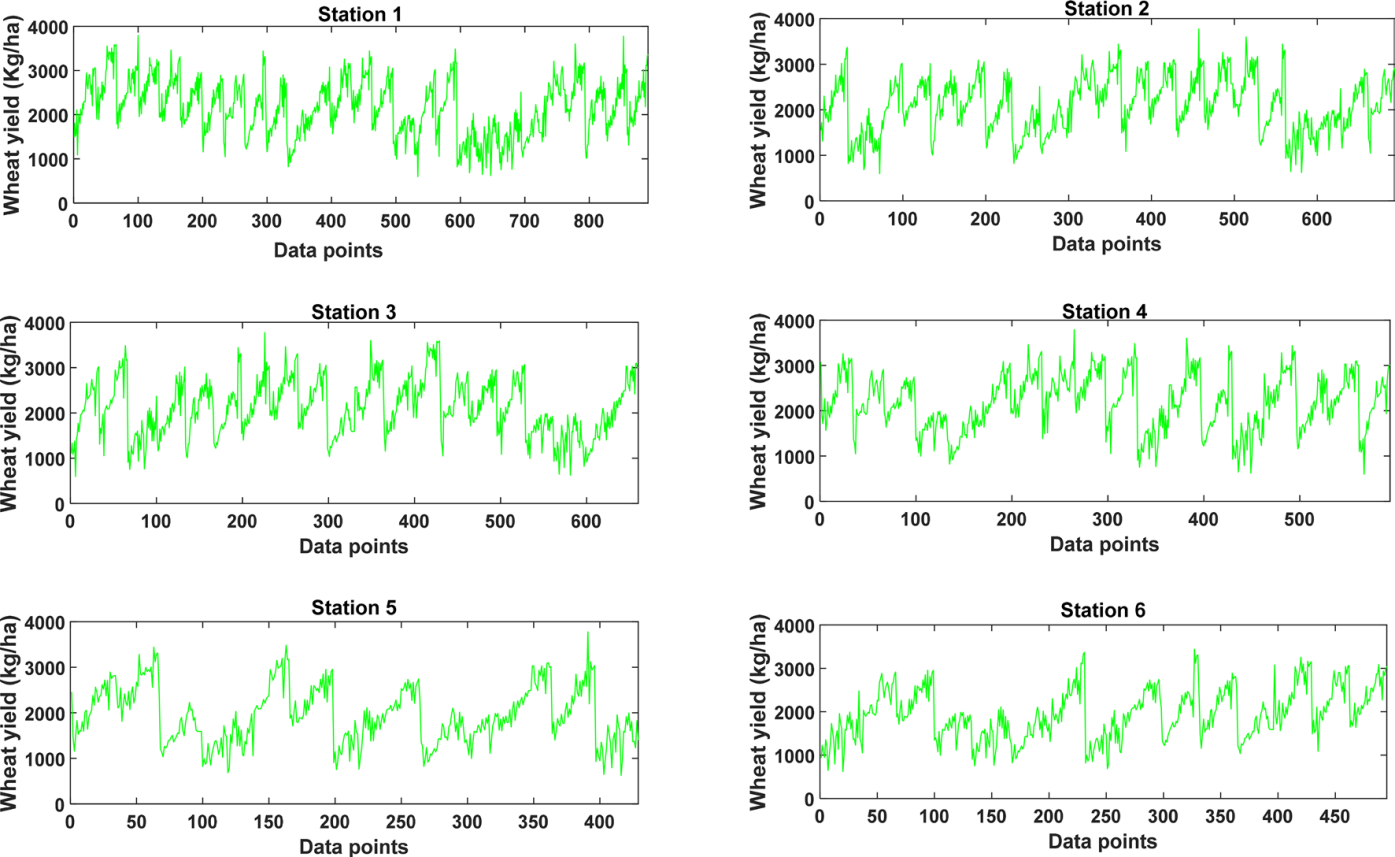
Time series of the annual wheat yield data for the training stations selected by the ant colony optimisation algorithm for each testing study station: Station 1: Rahimyar Khan, Station 2: D. G. Khan, Station 3: Kasur, Station 4: Sialkot, Station 5: Rawalpindi, and Station 6: Jhang.

In Eq. (9) $W$ indicates input/output of the wheat yield data, ${W}_{min}$ is the smallest value, ${W}_{max}$ is the largest value of wheat yield in the dataset, and ${W}_{norm}$ is the desired normalized value. The normalization process overcomes data fluctuations caused by inherent data features/patterns^77^. It essentially is invertible and in no way affects the results^77^. Figure 4 presents the time series of the tested study stations constructed from the selected features using the ACO algorithm.

#### Phase 2

**Figure 5:**
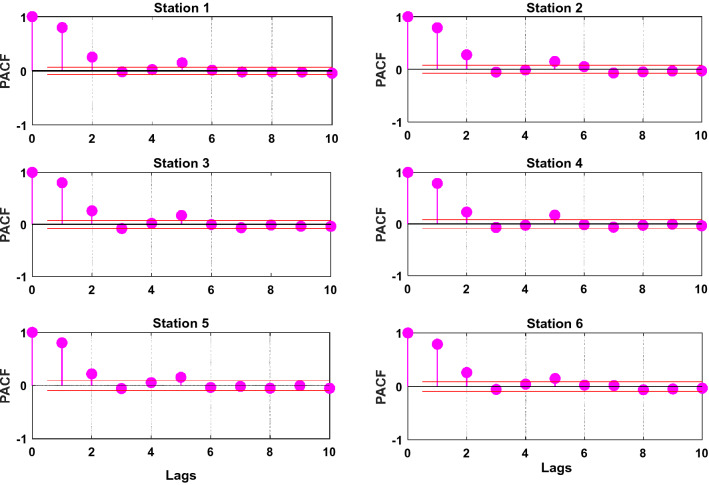
Partial autocorrelation function correlation coefficient (PACF) of the historical annual wheat yield time series for each testing study station: Station 1: Rahimyar Khan, Station 2: D. G. Khan, Station 3: Kasur, Station 4: Sialkot, Station 5: Rawalpindi, and Station 6: Jhang.

The partial autocorrelation function (*PACF*) was employed to calculate and determine the statistically significant lags of historical wheat yield data series as in Fig. 5. Moreover, the cross-validation or any data randomized approach cannot be adopted as time-series data by definition occur in a temporal order/sequence and this order or sequence must be preserved in order to keep the structure of the series intact^78^.

**Figure 6:**
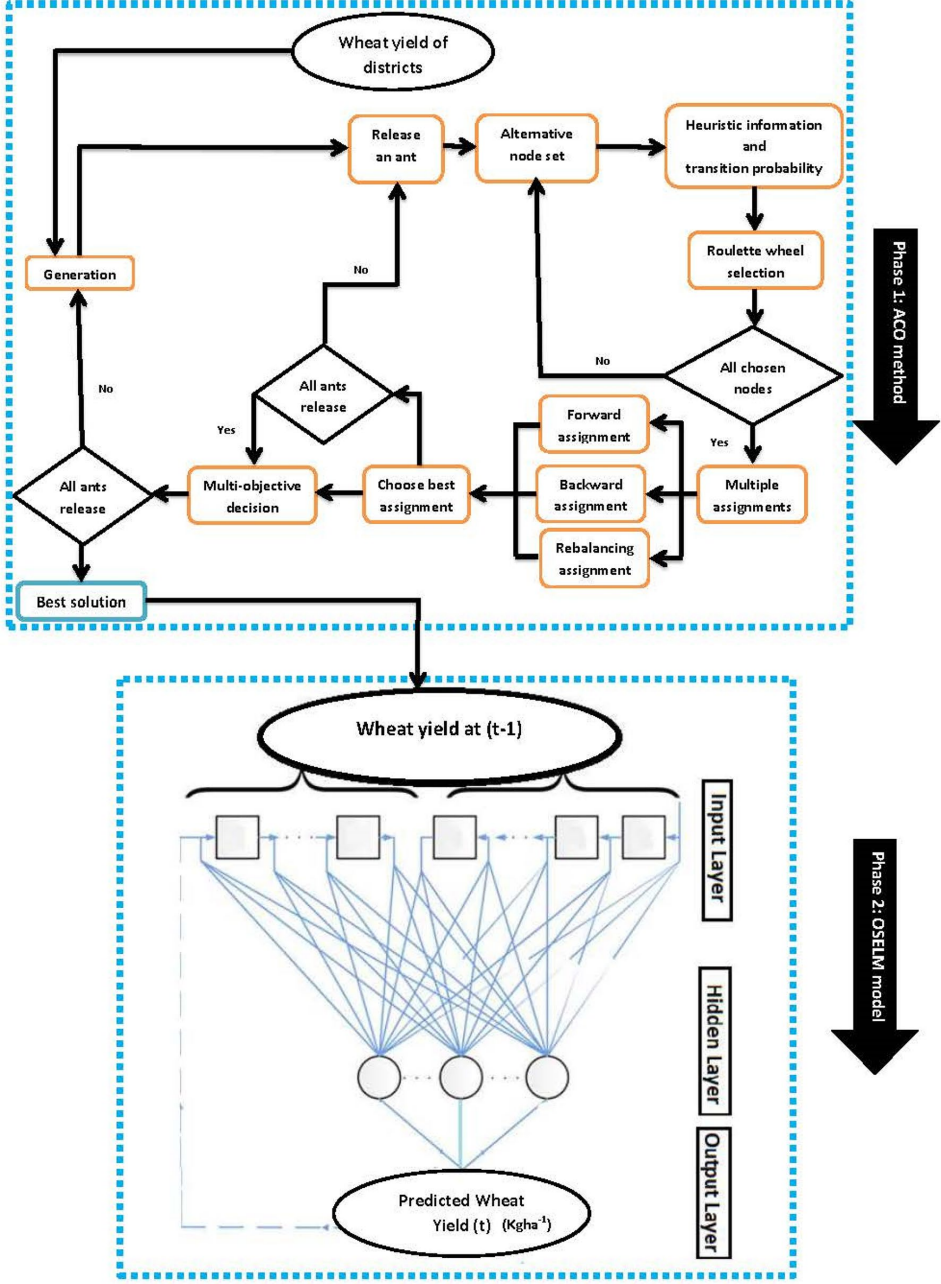
Flow chart of the proposed hybrid two-phase Ant Colony Optimization algorithm integrated with the Online Sequential Extreme Learning Machine (OSELM) model.

These significant lagged inputs at (*t* *−* 1) were incorporated as the input predictor in the OSELM model to forecast the yield W. Different activation functions (sigmoid, sine, hardlim, radial basis) were tested to determine the best activation function and the radial basis (*rbf*) and sigmoid (*sig*) functions were found to be the optimal ones. Consequently, different combinations of hidden neuron ranging from 7 to 35 were trialed with block size being fixed at 100. The second significant lag (*t* − 2) was also utilized in the proposed two-phase hybrid ACO-OSELM model to check whether model performance increases or not. But upon utilizing the lag (*t* − 2), the accuracy of the proposed two-phase hybrid ACO-OSELM model decreased, so it was not considered in this paper. Similarly, the benchmark models ELM and RF models were developed resulting in ACO-ELM and ACO-RF models respectively. Figure 6 displays the model schematics. Then model training performances of the proposed hybrid two-phase ACO-OSELM were assessed via correlation coefficient (*r*) and root-mean-squared-error (*RMSE*) as shown in Table 4.

**Table 4:** Training performance of two-phase hybrid ant colony optimization algorithm coupled online sequential extreme learning machines **(ACO-OSELM)** versus ACO-ELM and ACO-RF models with correlation coefficient (*r*) and root mean squared error (*RMSE*, kg ha^−1^).

Testing stations	Lags	ACO-OSELM		ACO-ELM		ACO-RF	
		Training period		Training period		Training period	
		*RMSE* (kg/ha)	*r*	*RMSE* (kg/ha)	*r*	*RMSE* (kg/ha)	***r***
Rahimyar Khan	W_t−1_	374.82	0.812	375.99	0.810	205.88	0.949
D. G. Khan	W_t−1_	381.57	0.790	382.11	0.790	212.60	0.942
Kasur	W_t−1_	370.49	0.804	366.78	0.808	201.27	0.948
Sialkot	W_t−1_	386.18	0.798	386.57	0.797	215.79	0.944
Rawalpindi	W_t−1_	356.80	0.832	357.93	0.831	213.64	0.946
Jhang	W_t−1_	353.55	0.799	352.46	0.799	214.46	0.933

The *r* and *RMSE* values attained during the training period by the two-phase hybrid ACO-OSELM models for wheat yield prediction at Rahimyar Khan and D. G. Khan were seen to be: *(r* = 0.812, 0.790, *RMSE* = 374.82, 381.57 kg ha^−1^). Equivalent metrics for Kasur and Sialkot were found to be: *(r* = 0.804, 0.798, *RMSE* = 370.49, 386.18 kg ha^−1^) and finally for Rawalpindi and Jhang were: *(r* = 0.832, 0.799, *RMSE* = 356.80, 353.55 kg ha^−1^). In addition, the training performances of comparative ACO-ELM and ACO-RF models were also studied (Table 4). The performance of the proposed two-phase hybrid ACO-OSELM model was relatively high during the training phase, and it is conjectured that the ACO-OSELM model accuracy in the testing phase for wheat yield prediction at these tested stations would be higher as well.

### Setting and tuning parameter optimization

To attain optimum precision, one of the most crucial tasks in designing the prediction model is to adjust the tuning and pruning of parameters associated with the models. Various approaches are adopted to fine-tune the parameters to acquire the desired optimum model. The trial and error strategy was utilized to get the optimum parameters during the constructing phase of the ACO-OSELM, ACO-ELM, and ACO-RF model to predict the wheat yield^79^. Table 5 presents the details of parameter settings during the prediction of annual wheat yield (*W*). The parameters utilized to design the ACO-OSELM model are the no. of hidden neurons, activation functions, and no. of blocks. The ACO-ELM model utilizes no. of hidden neurons and activation functions only, while ACO-RF requires two parameters: no. of trees and no. of split predictors. The details on fine-tuning of these parameters are provided in Table 5 for optimally performing ACO-OSELM, ACO-ELM, and ACO-RF model for all selected study stations.

**Table 5:** Tuning parameters of the ACO-OSELM, ACO-ELM and ACO-RF models to predict wheat yield.

Test stations	Models		Tuning parameter models
Rahimyar Khan	OSELM-ACO	W_t+1_	No. of hidden neuron = 35, activation function = rbf, no. of blocks = 100
	ELM-ACO	W_t+1_	Neuron = 15, activation no. of hidden function = sig
	RF-ACO	W_t+1_	No. of trees = 10,000, no. of split predictors = 2
D. G. Khan	OSELM-ACO	W_t+1_	No. of hidden neuron = 11, activation function = rbf, no. of blocks = 100
	ELM-ACO	W_t+1_	No. of hidden neuron = 15, activation function = sig
	RF-ACO	W_t+1_	No. of trees = 10,000, no. of split predictors = 2
Kasur	OSELM-ACO	W_t+1_	No. of hidden neuron = 7, activation function = rbf, no. of blocks = 100
	ELM-ACO	W_t+1_	No. of hidden neuron = 17, activation function = rbf
	RF-ACO	W_t+1_	No. of trees = 10,000, no. of split predictors = 2
Sialkot	OSELM-ACO	W_t+1_	No. of hidden neuron = 15, activation function = rbf, no. of blocks = 100
	ELM-ACO	W_t+1_	No. of hidden neuron = 9, activation function = rbf
	RF-ACO	W_t+1_	No. of trees = 10,000, no. of split predictors = 2
Rawalpindi	OSELM-ACO	W_t+1_	No. of hidden neuron = 35, activation function = rbf, no. of blocks = 100
	ELM-ACO	W_t+1_	No. of hidden neuron = 17, activation function = rbf
	RF-ACO	W_t+1_	No. of trees = 10,000, no. of split predictors = 2
Jhang	OSELM-ACO	W_t+1_	No. of hidden neuron = 10, activation function = sig, no. of blocks = 100
	ELM-ACO	W_t+1_	No. of hidden neuron = 15, activation function = sin
	RF-ACO	W_t+1_	No. of trees = 10,000, no. of split predictors = 2

### Model performance evaluation

Performance evaluations of the proposed hybrid two-phase ACO-OSELM versus ACO-ELM and the ACO-RF models applied for yield, *W*, forecasting was carried out via statistical standardized metrics and diagnostic plots^80^. These assessment metrics are formulated below as per^81–86^:

i. Correlation coefficient (*r*):
  10 $$r=\left(\frac{{\sum }_{i=1}^{N}\left({W}_{obs,i}-{\stackrel{\_}{W}}_{obs,i}\right)\left({W}_{pred,i}-{\stackrel{\_}{W}}_{pred,i}\right)}{\sqrt{{\sum }_{i=1}^{N}{\left({W}_{obs,i}-{\stackrel{\_}{W}}_{obs,i}\right)}^{2}}\sqrt{{\sum }_{i=1}^{N}{\left({W}_{pred,i}-{\stackrel{\_}{W}}_{pred,i}\right)}^{2}}}\right)$$
ii. Willmott’s index (*WI*):
  11 $$WI=1-\left[\frac{{\sum }_{i=1}^{N}{\left({W}_{pred,i}-{W}_{obs,i}\right)}^{2}}{{\sum }_{i=1}^{N}{\left(\left|{W}_{pred,i}-{\stackrel{\_}{W}}_{obs,i}\right|+\left|{W}_{obs,i}-{\stackrel{\_}{W}}_{obs,i}\right|\right)}^{2}}\right],\quad 0\le WI\le 1$$
iii. Nash–Sutcliffe coefficient (*NS*_*E*_):
  12 $$N{S}_{E}=1-\left[\frac{{\sum }_{i=1}^{N}{\left({W}_{obs,i}-{W}_{pred,i}\right)}^{2}}{{\sum }_{i=1}^{N}{\left({W}_{obs,i}-{\overline{W}}_{pred,i}\right)}^{2}}\right],\quad 0\le N{S}_{E}\le 1$$
iv. Root mean square error (*RMSE*, kg ha^−1^):
  13 $$RMSE=\sqrt{\frac{1}{N}\sum _{i=1}^{N}{\left({W}_{pred,i}-{W}_{obs,i}\right)}^{2}}$$
v. Mean absolute error (*MAE*, kg ha^−1^):
  14 $$MAE=\frac{1}{N}\sum _{i=1}^{N}\left|\left({W}_{pred,i}-{W}_{obs,i}\right)\right|$$
vi. Legates–McCabe’s (*LM*) index:
  15 $$LM=1-\left[\frac{{\sum }_{i=1}^{N}\left|{W}_{pred,i}-{W}_{obs,i}\right|}{{\sum }_{i=1}^{N}\left|{W}_{obs,i}-{\stackrel{\_}{W}}_{obs,i}\right|}\right],0\le LM\le 1$$
vii. Relative root mean square error (*RRMSE*, %):
  16 $$RRMSE=\frac{\sqrt{\frac{1}{N}{\sum }_{i=1}^{N}{\left({W}_{pred,i}-{W}_{obs,i}\right)}^{2}}}{\frac{1}{N}{\sum }_{i=1}^{N}\left({W}_{obs,i}\right)}\times 100$$
viii. Relative mean absolute percentage error (*RMAE*; %):
  17 $$RMAE=\frac{1}{N}\sum_{i=1}^{N}\left|\frac{\left({W}_{pred,i}-{W}_{obs,i}\right)}{{W}_{obs,i}}\right|\times 100$$

where ${W}_{obs,i}$ and ${W}_{pred,i}$ are *i*th observed and predicted values of the wheat yield *W*; ${\stackrel{\_}{W}}_{obs,i}$ and ${\stackrel{\_}{W}}_{pred,i}$ represents respective observed and predicted averages of *W* while *N* is the number of data points in the testing phase. The value of correlation coefficient (*r*) can be in the range of − 1 and + 1, which demonstrates the associations in terms of the proportion of variance in between the observed and predicted *W* from the machine learning models^82^. A value of + 1 shows that the observed and forecasted values are highly correlated with the least variances. The second metric Willmott’s Index *(WI)* ranges between 0 and 1. The *WI* overcomes the insensitivity issues as the differences between the observed and forecasted values are not squared and the ratio of the mean squared error in place of the differences are considered in computations^87,88^. The next metric, i.e., Nash–Sutcliffe Efficiency (*NS*_*E*_) has the ideal value of 1 and spans till negative infinity that essentially compares the variance of predicted with the observed values^89^. In addition, the computation of error measures *RMSE* and *MAE* are based on the aggregation of residuals of observed and predicted *W* values^90^. The higher *W* values are largely captured by the *RMSE* whereas the *MAE* equally assesses all variations regardless of the sign, yet both range from 0 (ideal value) to positive infinity. On the other hand, the Legates–McCabe’s (*LM*) index is a more robust tool developed to address the limitations of both the *W* and *NS*_*E*_ and the value is bound between 0 and 1 (the ideal value)^91^.

However, these metrics should not essentially be used to compare model performance at geographically diverse stations^92^, as these metrics are in their absolute terms. As such the relative values of root mean square error (*RRMSE*) and mean absolute error (*RMAE*) were utilized for this purpose^93^. The relative values are in percentages and for a model to be rated as outstanding, the (*RRMSE, RMAPE*) < 10%. For models rated as good the range is 10% < (*RRMSE, RMAE*) < 20%, while the model is fair if 20% < (*RRMSE, RMAE*) < 30% and if the (*RRMSE, RMAE*) > 30% the model is considered to have poor prediction performance^94,95^.

## Modelling results and analysis

**Figure 7:**
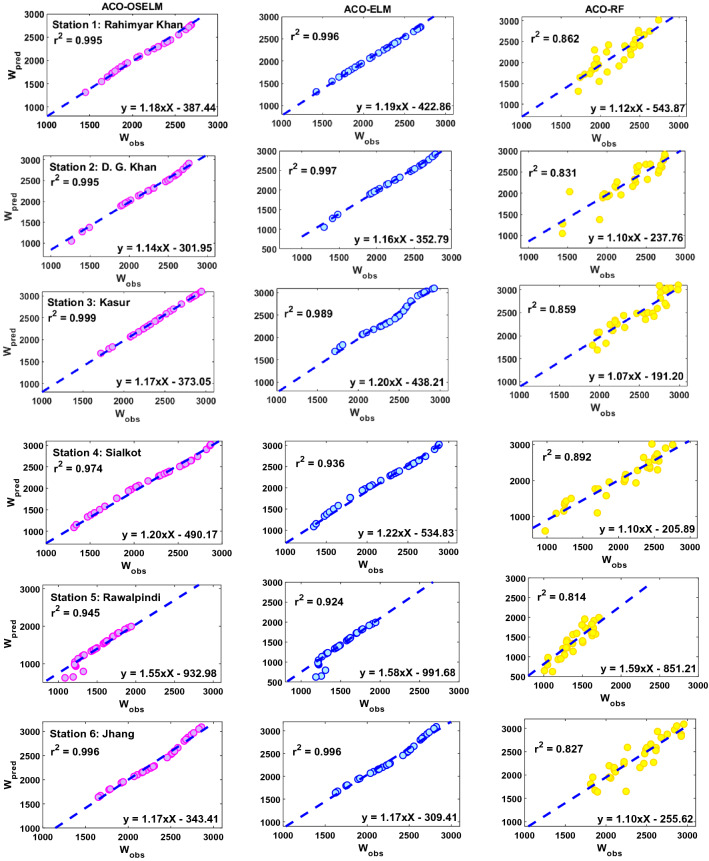
Scatterplots of the predicted (W_pred_) and observed wheat yield (W_obs_) (kg ha^−1^) in the testing phase of the **ACO-OSELM** versus ACO-ELM and ACO-RF models including the coefficient of determination (*r*^2^) and a linear fit inserted in each panel for the tested study zones. *Note*: Each point represents each year’s data in the testing period.

The proposed two-phase hybrid ACO-OSELM is evaluated with ACO-ELM and ACO-RF models, based on evaluation metrics (“Setting and tuning parameter optimization” section), diagnostic plots together with error distributions. Figure 7 displays a scatterplot with the respective coefficients of determination (*r*^2^) depicting the level of associations between the predicted and observed wheat yield (*W*) overlayed with the goodness-of-fit line and the linear equation. Essentially, the closer the linear equation is to the *y* = m*x* representation and the closer the *r*^2^ is to unity, the better the model performance is. The proposed two-phase hybrid ACO-OSELM model has better predictive potential than ACO-ELM and ACO-RF models in terms of *r*^2^ (ACO-OSELM ≈ 0.995, ACO-ELM ≈ 0.996, ACO-RF ≈ 0.862) for Kasur. Again, the proposed two-phase hybrid ACO-OSELM model is more accurate for Sialkot, *r*^2^ (ACO-OSELM ≈ 0.974, ACO-ELM ≈ 0.936, ACO-RF ≈ 0.892), and Rawalpindi stations in terms of the achieved *r*^2^ (ACO-OSELM ≈ 0.945, ACO-ELM ≈ 0.924, ACO-RF ≈ 0.814). The proposed two-phase hybrid ACO-OSELM model for other stations Rahimyar Khan, D. G. Khan, and Jhang is reasonably good compared to ACO-ELM and ACO-RF models (Fig. 7). The better accuracy of the proposed two-phase hybrid ACO-OSELM model against the comparison models for all the study regions is confirmed by the linear regression equation and the goodness-of-fit in addition to attaining larger *r*^2^ values.

**Figure 8:**
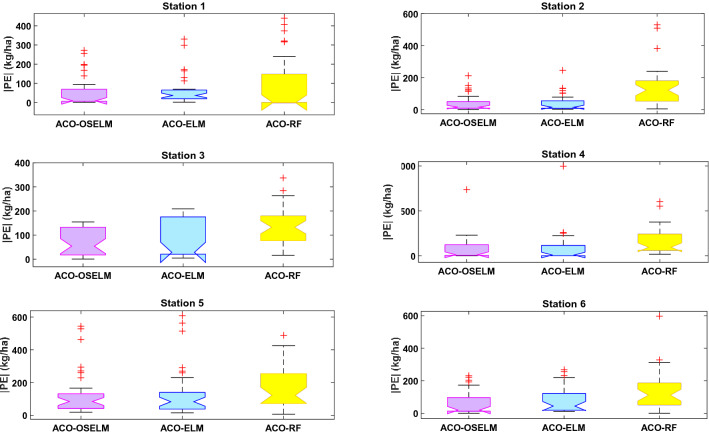
Boxplots of the prediction error |PE| (kg ha^−1^) of ACO-OSELM versus ACO-ELM and ACO-RF models between the predicted and observed wheat yield for Station 1: Rahimyar Khan, Station 2: D. G. Khan, Station 3: Kasur, Station 4: Sialkot, Station 5: Rawalpindi, and Station 6: Jhang.

Moreover, comparative boxplots of the proposed two-phase hybrid ACO-OSELM model with ACO-ELM and ACO-RF models for each station were established. Figure 8 displays these boxplots of absolute values of prediction error |PE| during the testing data together with respective upper quartiles, medians, and lower quartiles. The ‘+’ on the figure denotes the extreme values of the |PE| distributions. Subsequently, much smaller quartile values registered by the proposed two-phase hybrid ACO-OSELM model for Rahimyar Khan and D. G. Khan followed by the ACO-ELM and ACO-RF models confirmed better predictive performances. The proposed two-phase hybrid ACO-OSELM model achieved improved accuracies for Rawalpindi and Jhang stations in relation to the benchmark models. Similarly, the proposed two-phase hybrid ACO-OSELM model performed well for Sialkot and Kasur stations in predicting wheat yield followed by the ACO-ELM and ACO-RF models. The boxplot in Fig. 8 clearly shows that the proposed two-phase hybrid ACO-OSELM model at all six stations outperformed the comparative models.

The preciseness of the proposed two-phase hybrid ACO-OSELM is further evaluated with comparative ACO-ELM and ACO-RF models based on *r*, *RMSE,* and *MAE* (Table 6). The proposed two-phase hybrid ACO-OSELM model at Kasur station registered the largest *r* with least *RMSE* and *MAE* (*r* ≈ 0.999, *RMSE* ≈ 85.42 kg ha^−1^,* MAE* ≈ 66.54 kg ha^−1^). In comparison, the ACO-ELM attained the following values (*r* ≈ 0.987, *RMSE* ≈ 111.59 kg ha^−1^,* MAE* ≈ 78.15 kg ha^−1^) while the ACO-RF model recorded; *r* ≈ 0.926, *RMSE* ≈ 154.36 kg ha^−1^,* MAE* ≈ 135.24 kg ha^−1^. Similarly, for Sialkot station, these metrics were ACO-OSELM (*r* ≈ 0.984, *RMSE* ≈ 155.86 kg ha^−1^, *MAE* ≈ 76.95 kg ha^−1^), followed ACO-ELM (*r* ≈ 0.967, *RMSE* ≈ 197.10 kg ha^−1^, *MAE* ≈ 83.21 kg ha^−1^) and ACO-RF (*r* ≈ 0.942, *RMSE* ≈ 209.89 kg ha^−1^, *MAE* ≈ 155.35 kg ha^−1^). Likewise, the performance of the proposed two-phase hybrid ACO-OSELM model was better for Rawalpindi, Jhang, Rahimyar Khan, and D. G. Khan in terms of registering the largest values of *r* and smallest *RMSE* and *MAE* values. This gives a clear indication of better performance of the proposed two-phase hybrid ACO-OSELM model, which can be considered a better data-intelligent tool for wheat yield prediction in contrast to the ACO-ELM and ACO-RF models.

**Table 6:** Testing performance of **ACO-OSELM** versus ACO-ELM and ACO-RF models measured by root mean square error (*RMSE*), mean absolute error (*MAE*), coefficient of determination (*r*).

Testing stations	Lags	ACO-OSELM			ACO-ELM			ACO-RF		
		*RMSE* (kg ha^−1^)	*MAE* (kg ha^−1^)	*r*	*RMSE* (kg ha^−1^)	*MAE* (kg ha^−1^)	*r*	*RMSE* (kg ha^−1^)	*MAE* (kg ha^−1^)	*r*
Rahimyar Khan	W_t−1_	94.96	56.16	0.996	99.06	63.26	0.997	212.32	175.25	0.929
D. G. Khan	W_t−1_	67.12	42.37	0.997	68.98	44.35	0.998	189.44	141.92	0.912
Kasur	W_t−1_	85.42	66.54	0.999	111.59	78.15	0.987	154.36	135.24	0.926
Sialkot	W_t−1_	155.86	76.95	0.984	197.10	83.21	0.967	209.89	155.35	0.942
Rawalpindi	W_t−1_	191.89	129.86	0.967	204.59	134.39	0.955	203.53	165.47	0.898
Jhang	W_t−1_	96.24	61.41	0.992	114.33	80.81	0.992	181.10	134.10	0.909

**Figure 9:**
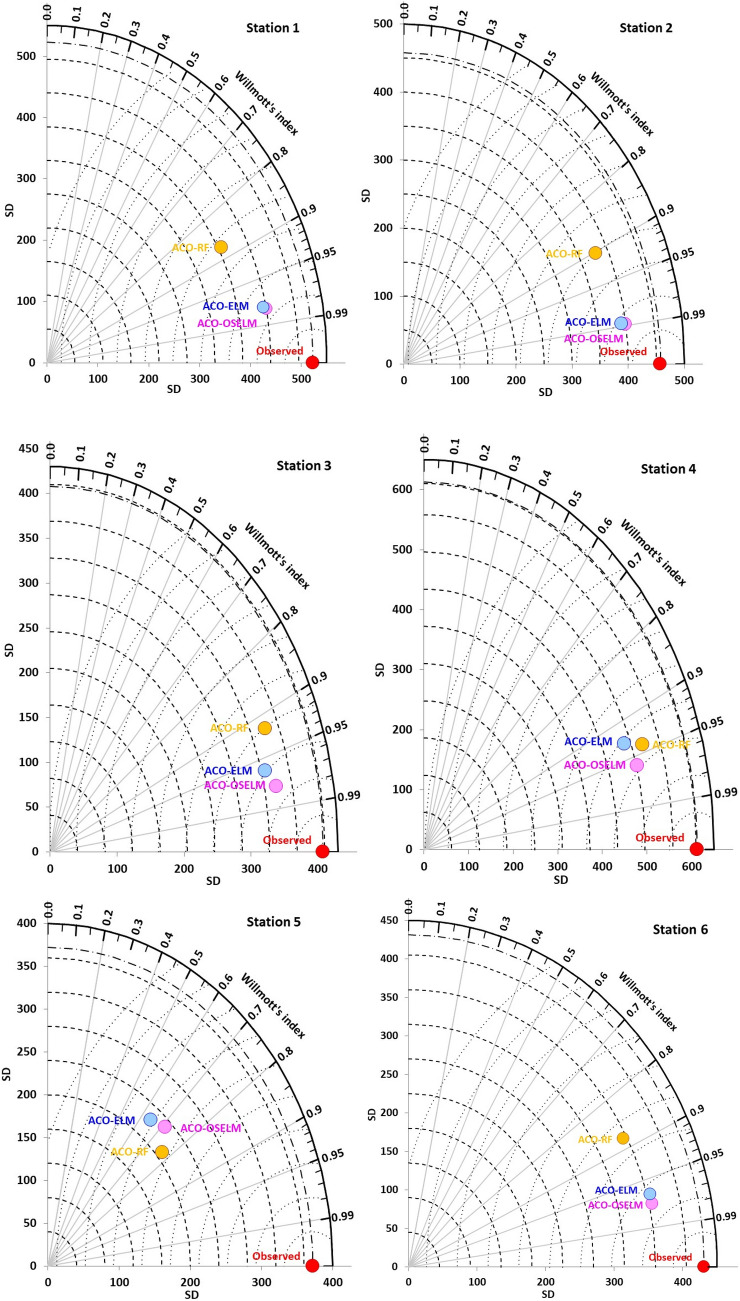
Vector field evaluation (VFE) diagram showing Willmott’s agreement between the observed and predicted wheat yield and standard deviation (SD) of **ACO-OSELM** versus ACO-ELM and ACO-RF models for all tested stations.

A vector field evaluation (VFE) diagram (Fig. 9) presents a more concise statistical summary of the associations of predicted and observed wheat yield matched based upon the respective *WI* values. A VFE diagram is a generalization of the Taylor diagram that provides an evaluation of model performances in terms of vector fields^96^. For Rahimyar Khan, the *WI* of the proposed two-phase hybrid ACO-OSELM model with observations was ~ 0.98, followed by ACO-ELM *≈* 0.97 and ACO-RF *≈* 0.87. The *WI* ~ 0.99 of the ACO-OSELM model was closest to the observed wheat yield as compared to ACO-ELM and ACO-RF for D. G. Khan stations. Similarly, the proposed two-phase hybrid ACO-OSELM models were found to be the best performing models for Kasur station (*WI* ≈ 0.98) that were within close proximity of the observed wheat yield followed by ACO-ELM (≈ 0.97) and ACO-RF (≈ 0.92) models. For other stations Sialkot and Jhang, the proposed two-phase hybrid ACO-OSELM model is closer to the observed *W* as compared to the ACO-ELM and ACO-RF models. For the Rawalpindi station, the ACO-RF model was marginally better than ACO-OSELM and ACO-ELM models. Overall, the *WI* of the proposed two-phase ACO-OSELM model was closely distributed to the observed baseline compared to the other models.

After that, the ACO-ELM and ACO-RF models were evaluated in terms of *WI, NS*_*E,*_ and *LM* for all candidate stations. The preciseness of the proposed two-phase hybrid ACO-OSELM model is presented in Table 7. The largest magnitudes of *WI* ≈ 0.980*, NS*_*E*_ ≈ 0.966, and *LM* ≈ 0.865 were recorded by the proposed two-phase hybrid ACO-OSELM model at Rahimyar Khan station followed by ACO-ELM (*WI* ≈ 0.978*, NS*_*E*_ ≈ 0.963 and *LM* ≈ 0.848) and the ACO-RF (*WI* ≈ 0.876*, NS*_*E*_ ≈ 0.830 and *LM* ≈ 0.579*)* models. For D. G. Khan and Kasur stations, again the proposed two-phase hybrid ACO-OSELM appeared to be the best model (*WI* ≈ 0.989, 0.977,* NS*_*E*_ ≈ 0.978, 0.955, and *LM* ≈ 0.884, 0.805), followed by ACO-ELM (*WI* ≈ 0.988, 0.962,* NS*_*E*_ ≈ 0.977, 0.923 and *LM* ≈ 0.879, 0.766) and ACO-RF models (*WI* ≈ 0.903, 0.920,* NS*_*E*_ ≈ 0.823, 0.852 and *LM* ≈ 0.612, 0.595). For other stations Sialkot, Rawalpindi, and Jhang, the proposed two-phase hybrid ACO-OSELM model appeared to be the best (Table 7) in comparison to the counterpart models revealing the better performances of the proposed two-phase hybrid ACO-OSELM models.

**Table 7:** The performance of **ACO-OSELM** versus ACO-ELM and ACO-RF models using Willmott’s index (*WI*), Nash–Sutcliffe (*NS*_*E*_*)* and Legates–McCabe’s (*LM*) agreement, for Station 1: Rahimyar Khan, Station 2: D. G. Khan, Station 3: Kasur, Station 4: Sialkot, Station 5: Rawalpindi and Station 6: Jhang.

Testing stations	Lags	ACO-OSELM			ACO-ELM			ACO-RF		
		*WI*	*NS*_*E*_	*LM*	*WI*	*NS*_*E*_	*LM*	*WI*	*NS*_*E*_	*LM*
Rahimyar Khan	W_t−1_	**0.980**	**0.966**	**0.865**	0.978	0.963	0.848	0.876	0.830	0.579
D. G. Khan	W_t−1_	**0.989**	**0.978**	**0.884**	0.988	0.977	0.879	0.903	0.823	0.612
Kasur	W_t−1_	**0.977**	**0.955**	**0.805**	0.962	0.923	0.766	0.920	0.852	0.595
Sialkot	W_t−1_	**0.960**	**0.933**	**0.845**	0.931	0.89	0.833	0.941	0.879	0.687
Rawalpindi	W_t−1_	**0.712**	**0.726**	**0.570**	0.647	0.688	0.555	0.772	0.692	0.453
Jhang	W_t−1_	**0.974**	**0.949**	**0.833**	0.966	0.927	0.781	0.883	0.818	0.636

Note that the best model is boldfaced (underline).

**Figure 10:**
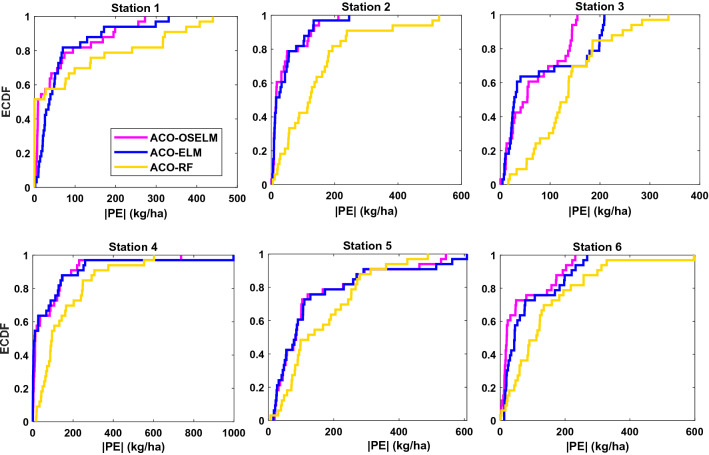
Empirical cumulative distribution function (ECDF) of the prediction error, |PE| (kg ha^−1^) for the testing stations using ACO-OSELM versus ACO-ELM and ACO-RF models.

Furthermore, the empirical cumulative distribution function (ECDF, Fig. 10) at all stations depicts that the proposed two-phase hybrid ACO-OSELM method was reasonably better and superior to both the ACO-ELM and ACO-RF models. Based on the error (0 to ± 400 kg ha^−1^) for the Rahimyar Khan, D. G. Khan, and Kasur station, (0 to ± 600 kg ha^−1^) for Rawalpindi and Jhang station while (0 to ± 1000 kg ha^−1^) for Sialkot station, Fig. 10 clearly proves that the proposed two-phase hybrid ACO-OSELM method was the most precise model in predicting wheat yield.

The magnitudes of relative root mean squared error (*RRMSE*) and relative mean absolute error (*RMAE*) for the different locations (Rahimyar Khan, D. G. Khan, Kasur, Sialkot, Rawalpindi, and Jhang) are presented in Table 8. It demonstrated that D. G. Khan is the station where the ACO-OSELM wheat yield predicting model performed the best with *RRMSE* ≈ 3.00 and *RMAE* ≈ 2.25%*.* The ACO-OSELM model was found to generate the least relative percentage error values (i.e., *RRMSE, RMAE*) at all tested stations except for the Rawalpindi station. However, the predicted errors generated by the proposed two-phase hybrid ACO-OSELM model were low in terms of their relative error values, and more importantly, the error values were within the recommended range of 10% threshold for an excellent model classification, except for Rawalpindi station^97^.

**Table 8:** Geographic comparison of the accuracy of the **ACO-OSELM** versus ACO-ELM and ACO-RF models in terms of relative root mean squared error (*RRMSE*, %) and the relative mean absolute error (*RMAE*, %) computed within the test stations.

Site and model data		ACO-OSELM		ACO-ELM		ACO-RF	
Testing stations	Lags	*RRMSE* (%)	*RMAE* (%)	*RRMSE* (%)	*RMAE* (%)	*RRMSE* (%)	*RMAE* (%)
Rahimyar Khan	W_t−1_	4.11	2.27	4.29	2.57	9.20	8.20
D. G. Khan	W_t−1_	**3.00**	**2.25**	3.09	2.39	8.48	7.33
Kasur	W_t−1_	3.42	2.42	4.47	2.79	6.19	5.59
Sialkot	W_t−1_	7.60	7.40	9.61	8.74	10.23	9.52
Rawalpindi	W_t−1_	13.96	13.95	14.88	14.70	14.80	14.42
Jhang	W_t−1_	4.09	2.26	4.86	3.06	7.70	6.09

Note that the best model is boldfaced (underline).

**Figure 11:**
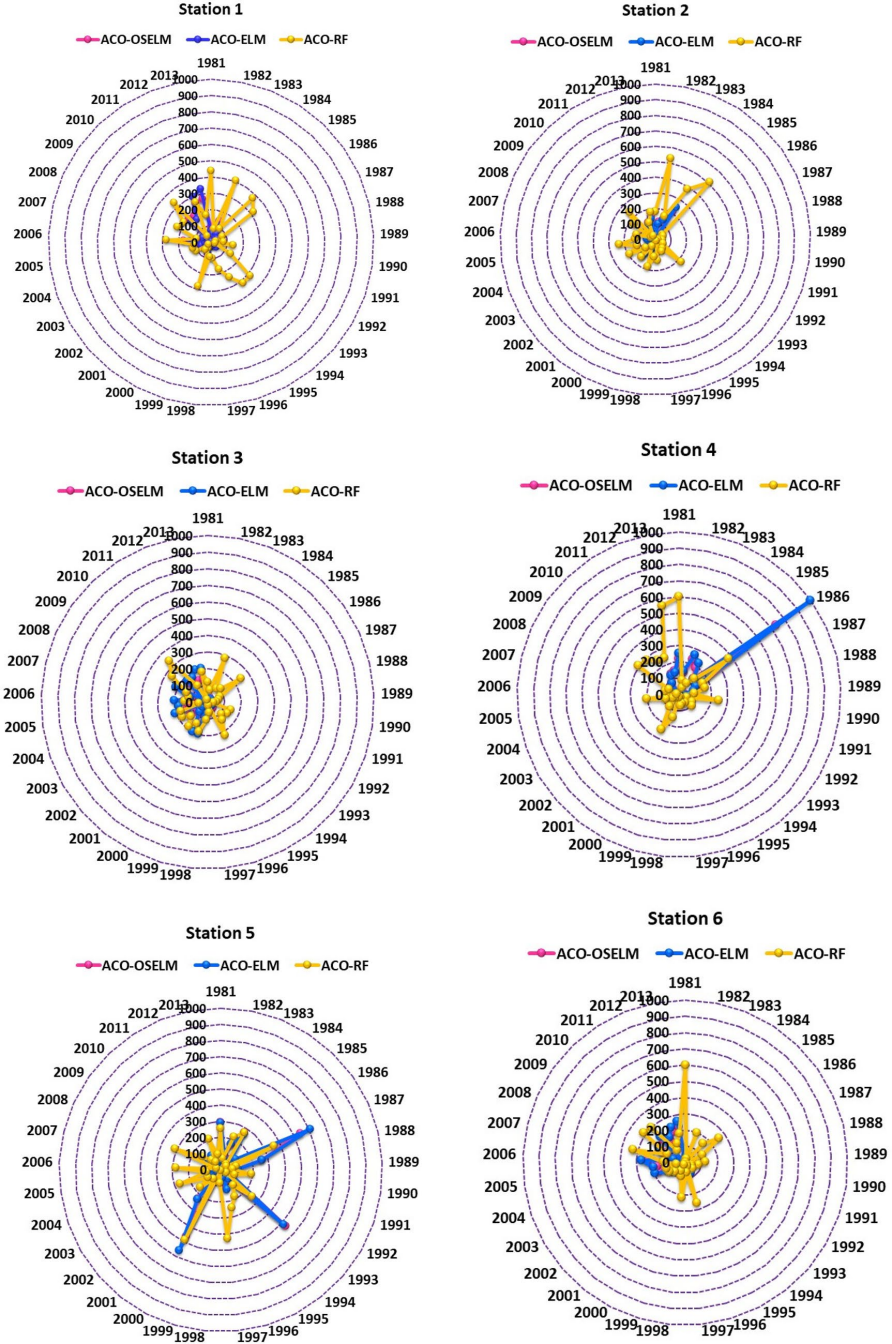
Polar plots showing the prediction error |PE| in each year generated from the **ACO-OSELM** versus ACO-ELM and ACO-RF models in predicting wheat yield for all stations.

Figure 11 presents the absolute prediction error |PE| in each year from 1981–2013 of the proposed two-phase hybrid ACO-OSELM versus ACO-ELM and ACO-RF models at the testing stations in the form of polar plots. For all stations, the prediction errors generated by the proposed two-phase hybrid ACO-OSELM were very low compared to the ACO-ELM and ACO-RF models. This was justified by the minimum values of relative prediction errors. The |PE| errors were significantly smaller in each year for the proposed two-phase hybrid ACO-OSELM model as compared to ACO-ELM and ACO-RF models in Rahimyar Khan, D. G. Khan, Kasur, Sialkot, Rawalpindi and Jhang stations. Overall, the proposed two-phase hybrid ACO-OSELM model generated better significant accuracy with smaller error statistics and higher *WI*.

## Discussions

Developing strategies for accurate crop yield prediction that can address food scarcity issues, decision-making on national imports and exports, and setting the prices in agriculture markets, can play an important role in policymaking, particularly in agriculture-based nations such as Pakistan. This study was aimed at designing a novel two-phase hybrid ACO-OSELM model using significant lag at (*t* *−* 1) to predict future wheat yield. This is a practically useful approach for crop yield management in terms of using the wheat yield data from several nearby stations in developing better agricultural practices and efficient precision agricultural technologies. For example, the methodology can be used in remote areas where meteorological data is not available due to limited resources. The research framework in this study can be applied to any other study station where yield data (whether it is wheat or any other crop) are available from surrounding stations to provide an accurate prediction.

The proposed two-phase hybrid ACO-OSELM model with its counterpart models (ACO-ELM and ACO-RF) was suitably evaluated, which revealed smaller relative percentage errors in terms of *RRMSE* and *RMAE* being generated. Respectively, reasonably large statistical correlation metric values of Legates–McCabe’s between predicted and observed yielded for D. G. Khan and other tested stations (Tables 7, 8). The model performances were high since the percentage errors achieved were less than 10%. Thus, the proposed two-phase hybrid ACO-OSELM model can suitably be used to predict the wheat yield where the prediction of a crop commodity is likely to become more important due to ever-increasing demand.

The proposed two-phase hybrid OSELM model can assist the government’s national policymakers and agricultural engineers in minimizing uncertainties in crop estimates reducing price hikes as well as unwarranted wastages^98^. Since the proposed two-phase hybrid ACO-OSELM model offers better forecasting potential together with being fast and robust, it can possibly be explored in predicting other crop yields including Rice, Maize, Cotton, Sugarcane, and Oilseeds to generate similar optimal predictions in follow-up studies.

The utilization of historical wheat yield data as inputs to predict the future yield carries some limitations. Certainly, weather conditions are a big driver for any agricultural yield. Hence, to enhance the scope of future studies, predictor inputs consisting of meteorological data (i.e., precipitation, air temperatures, soil moisture, wind speed, solar radiation, etc.) need to be used to predict future crop yield as crop production amounts are largely contingent upon these parameters. Satellite-based remotely sensed data and/or data from atmospheric simulation models (e.g.,^31,99,100^) as predictor variables are also likely to add great value to the crop yield modeling in remote agricultural areas with limited datasets. Incorporation of remotely sensed photosynthetically active radiation (PAR) that reveals crop health could also be focussed on, in an independent study. In addition, fertilizer/manure usage data accompanied by relevant soil characteristics (e.g., texture, pedality, hydraulics, porosity, bulk density, thickness, and soil organic carbon content) could also be explored in the proposed two-phase hybrid ACO-OSELM model. Fields that use irrigation for production need to utilize irrigation statistics to improve crop yield predictions. Further, as process-based modeling is resource-demanding which emerging agricultural nations like Pakistan unable to afford, the proposed two-phase hybrid ACO-OSELM model could be used as a feasible option.

An ensemble modeling approach could further improve the two-phase hybrid ACO-OSELM modeling with the possibility of achieving better results. Ensemble modeling would provide better confidence in predictions making the model more reliable in strategic decision-making, as uncertainties between several forecasted data would be captured and displayed in the outputs. Quantum-Behaved PSO and the Firefly Algorithms could also be used to identify training stations that have been tested to hybridize with the OSELM (e.g.,^101,102^). Future works could apply, empirical wavelet transform-EWT^103^, empirical mode decomposition-EMD^104^, and singular value decomposition-SVD^105^, as data pre-processing tools in modeling and predicting crop yields.

## Conclusions

The current study was adopted to develop a robust two-phase hybrid ACO-OSELM model to predict wheat yield. Lagged wheat yields from several neighbouring stations were used for training purposes as model predictors for the candidate station. Wheat yield data for the period of 1981–2013 from 26 stations were pooled and the best training stations were selected by the ACO algorithm on the basis of feature selection corresponding to the 27th test station. The selected feature stations were used to construct a time series where the significant lags at (*t* − 1) were used to develop the proposed two-phase hybrid ACO-OSELM model to achieve better accuracy. Several evaluation criteria including diagnostic plots were adopted to judge the accuracy of the proposed two-phase hybrid ACO-OSELM model. The proposed hybrid ACO-OSELM outperformed the counterpart models for wheat yield prediction. The prediction errors metrics for the best station D. G. Khan registered by ACO-OSELM model were *RMSE* ≈ 67.12 kg ha^−1^ and *MAE* ≈ 42.37 kg/ha. The normalized performance metrics for the D. G. Khan station (*r* ≈ 0.997, *WI* ≈ 0.989 and *NS*_*E*_ ≈ 0.978. The performance assessment of the ACO-OSELM model in relation to ACO-ELM and the ACO-RF models via Legates–McCabe’s indices were in agreement. The *LM* values between the predicted and observed wheat yield for the D. G. Khan study station were *LM* ≈ 0.884 (ACO-OSELM), 0.879 (ACO-ELM) and 0.612 (ACO-RF), respectively and the relative errors, *RRMSE* and *RMAE* were very small: 3.00%, 2.25% (ACO-OSELM) compared with 3.09%, 2.39% (ACO-ELM) and 8.48%, 7.33% (ACO-RF). Since the relative percentage errors, *RRMSE* and *RMAE* showed that at D. G. Khan station the proposed two-phase hybrid ACO-OSELM model performed the best as compared to other stations, evidently geographic variability does influence the outcomes to a certain degree. This essentially is a baseline study whereby wheat yield data from several stations are being utilized to predict wheat yield more accurately that can potentially be extended to forecasting using other climatological parameters in future studies. Similarly, other agricultural crop yield predictions could be explored with the proposed two-phase hybrid ACO-OSELM model that will assist policymakers and decision-makers in the better management of crop yield and price predictions. More importantly, accurate wheat and other crop yield predictions can be used to alert impacted stakeholders and the government to avert food security issues.
